# Clinical Characteristics of Korean Breast Cancer Patients Who Carry Pathogenic Germline Mutations in Both BRCA1 and BRCA2: A Single-Center Experience

**DOI:** 10.3390/cancers12051306

**Published:** 2020-05-21

**Authors:** Joon Young Hur, Ji-Yeon Kim, Jin Seok Ahn, Young-Hyuck Im, Jiyun Lee, Minsuk Kwon, Yeon Hee Park

**Affiliations:** 1 Division of Hematology-Oncology, Department of Medicine, Samsung Medical Center, Sungkyunkwan University School of Medicine, Seoul 06351, Korea; md.joon@gmail.com (J.Y.H.); jyeon25.kim@samsung.com (J.-Y.K.); ajis@skku.edu (J.S.A.); imyh00@skku.edu (Y.-H.I.); jiyunlee90@gmail.com (J.L.); minsuk.alex.kwon@gmail.com (M.K.); 2 Division of Hematology and Oncology, Department of Internal Medicine, Hanyang University Guri Hospital, Guri 11923, Korea

**Keywords:** BRCA1, BRCA2, breast cancer

## Abstract

There are few reports of breast cancer patients who carry germline mutations in both germline breast cancer susceptibility genes 1 (gBRCA1) and 2 (gBRCA2). In this study, we analyzed the clinical, pathological, and genomic characteristics of Korean breast cancer patients with both gBRCA1 and gBRCA2 mutations. Medical records of patients who received gBRCA1 and gBRCA2 testing at Samsung Medical Center between January 2007 to October 2018 were retrospectively reviewed. Genomic DNA was isolated from peripheral blood leukocytes. Among a total of 2720 patients, four patients with both gBRCA1 and gBRCA2 mutations were identified (4/2720; 0.14%). Seven patients who had a gBRCA1 mutation and gBRCA2 variants of uncertain significance (VUS) were also identified. In those patients with both gBRCA1 and gBRCA2 mutations, the mean age at diagnosis for breast cancer was 36 years (range, 31–43 years). All four tumors were infiltrating ductal carcinomas and three of the tumors were estrogen receptor-negative, progesterone receptor-negative, and human epidermal growth factor receptor 2-negative (triple-negative). All four patients who carried germline mutations in both BRCA1 and BRCA2 had a family history of breast/ovarian cancer. Pathologic stage was II in three patients and I in one patient. Breast cancer patients with both gBRCA1 and gBRCA2 mutations were rare, young at diagnosis, and all but one tumor was triple-negative based on our single-center experience.

## 1. Introduction

Most cases of breast cancer are not hereditary, but breast cancer without an inherited component appears both sporadically and in multiply affected families [1]. Both breast cancer susceptibility genes (BRCA)1 and BRCA2 proteins are critical to the repair of double-strand DNA breaks due to their function in homologous recombination repair (HRR), a form of DNA repair that uses a homologous DNA sequence to guide repair at double-stranded DNA breaks [2]. Mutations in these genes are considered to be responsible for approximately 40% of familial breast cancers and for the majority of familial ovarian cancers, and account for 5% to 20% of all breast and ovarian cancers [3]. Germline mutations in BRCA1 confer a cumulative breast and ovarian cancer risk by age 80 of 72% and 44%, respectively, while BRCA2 mutations confer a 69% and 17% increased risk of breast and ovarian cancer by this age, respectively [4]. BRCA1 is ubiquitously expressed, and it remains a mystery why BRCA1 mutation leads specifically to breast and ovarian cancer. BRCA2 mutation is associated with increased risks of pancreatic cancer and high-grade prostate cancer [1].

Mutations in BRCA1 and BRCA2 are found in approximately 1 in 300 individuals in the general population and 1 in 40 individuals of Ashkenazi Jewish descent [5]. In the Korean hereditary breast cancer (KOHBRA) study, the incidence of BRCA mutation was found to be 24.8% (106/428) in breast cancer patients with a family history of breast/ovarian cancers [6]; However, few studies have investigated breast cancer patients who carry germline mutations in both BRCA1 and BRCA2. In this study, we describe the clinical, pathological, and genomic characteristics of Korean breast cancer patients with germline mutations of both BRCA1 and 2 based on a single-center experience. 

## 2. Results

### 2.1. Clinical Characteristics of Patients with Pathogenic Germline BRCA1 and 2 Mutations

**Figure 1:**
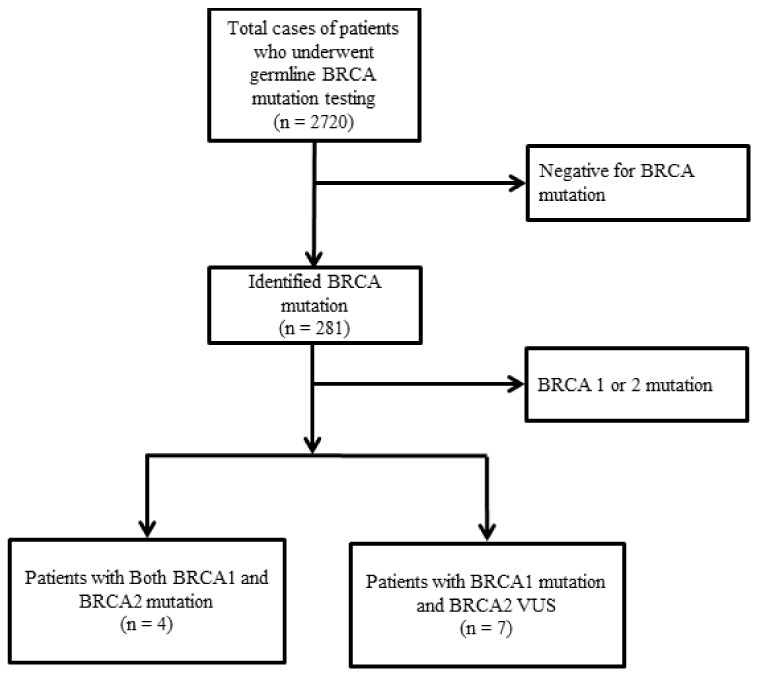
Flow chart of patient selection. Among a total of 2720 patients, four patients with both germline breast cancer susceptibility genes (gBRCA) 1 and 2 mutations were identified (4/2720; 0.14%). There were seven patients with gBRCA1 mutation and gBRCA2 variants of unknown significance (VUS), but no patients with gBRCA2 mutation and gBRCA1 VUS. BRCA, breast cancer susceptibility genes; VUS, variant of uncertain significance.

Among a total of 2720 patients, four patients with both germline BRCA (gBRCA)1 and gBRCA2 mutations were identified (4/2720; 0.14%) (Figure 1). All patients were female and the mean age at diagnosis for breast cancer was 36 years (range, 31–43 years). All four tumors were infiltrating ductal carcinomas. Two patients received breast-conserving surgery, while the other two received a total mastectomy due to bilateral breast cancer. Pathologic stage was II in three tumors and I in the remaining tumor. All patients (*n* = 4) were tested for BRCA mutations because of a familial history of breast/ovarian cancer. Three of the patients were married and had given birth. Clinical characteristics of patients with germline mutations in both BRCA1 and BRCA2 are described in Table 1.

### 2.2. First Case

**Figure 2:**
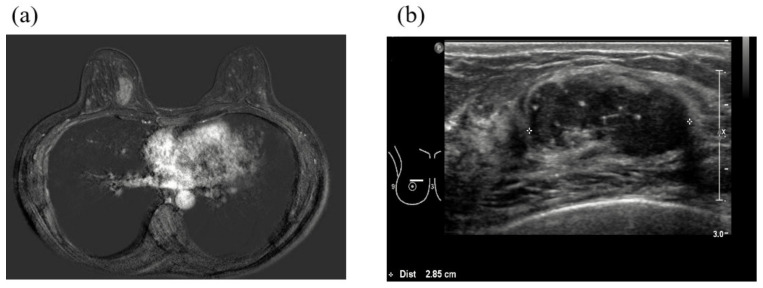
Imaging of patient No. 1 at the first diagnosis of breast cancer in January 2016. (**a**) Breast magnetic resonance imaging (MRI) showed a 2.4 cm lesion in the right breast. (**b**) Breast ultrasound image showed a hypoechoic solid lesion in the right breast.

**Figure 3:**
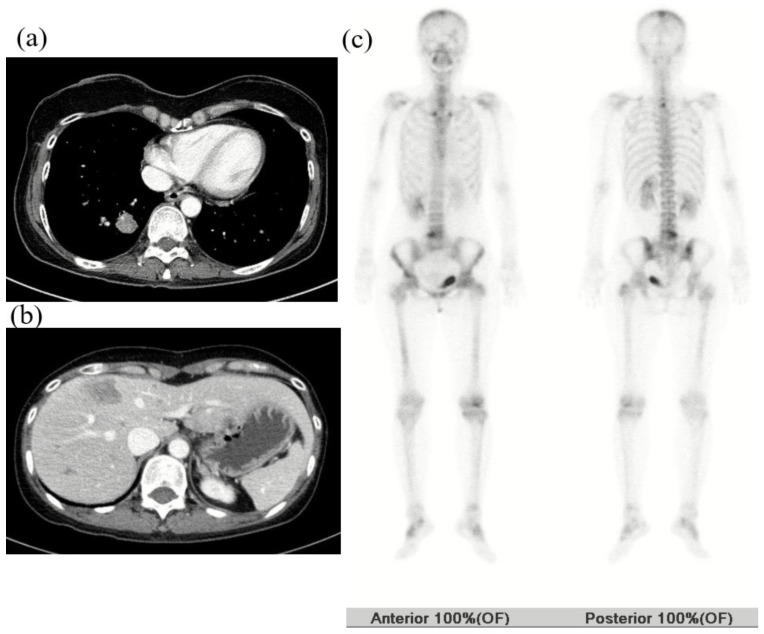
Imaging of patient No. 1 at the first recurrence of breast cancer in September 2018. (**a**,**b**) Computed tomography (CT) scan of the chest and abdomen showed a metastatic mass in the right lower lobe of the lung and multiple hepatic masses. (**c**) Body bone scan revealed multiple metastatic bone lesions (left frontal bone, thoracic spine, right humeral shaft, and left proximal femur).

**Figure 4:**
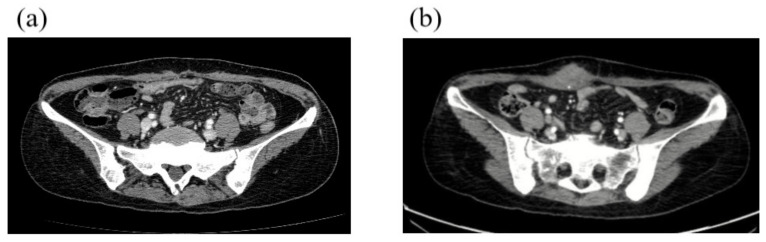
Imaging of patient No 1. in 2019. (**a**) Computed tomography (CT) scan of the pelvis showed a metastatic lesion in the rectus muscle in January 2019. (**b**) The metastatic lesion in the rectus muscle had increased in size by March 2019.

Patient No. 1, a premenopausal 43-year-old woman, developed right breast cancer (invasive ductal carcinoma, stage T2N0M0, Figure 2) that was estrogen receptor (ER)-negative, progesterone receptor (PR)-negative, and human epidermal growth factor receptor 2 (HER2)-negative (i.e., triple-negative) in January 2016. Serum cancer antigen 15-3 (CA15-3) was 18 IU/mL (normal <28 IU/mL). She underwent neoadjuvant chemotherapy (doxorubicin and cyclophosphamide followed by docetaxel) followed by breast-conserving surgery and adjuvant radiotherapy. She underwent BRCA1 and BRCA2 testing because her mother had a history of breast cancer. Sequence analysis showed a base substitution at position 390 of BRCA1 that generated a stop codon (p.Tyr130Ter). A frame shift mutation was found in BRCA2 at the amino acid position 1859 (p.Ile1859fs) (Table 2). This patient underwent preventive salpingo-oophorectomy and right preventive mastectomy. Triple-negative breast cancer (TNBC) recurred due to multiple hepatic masses, a metastatic mass in the right lower lobe of the lung, and multiple metastatic bone lesions (left frontal bone, thoracic spine, right humeral shaft, and left proximal femur) in September 2018 (Figure 3). She underwent palliative radiotherapy to the femur and palliative chemotherapy (TC, paclitaxel, and carboplatin). Three months after the initiation of palliative treatment, the disease had progressed to the rectus muscle (Figure 4). The patient died due to the progression of her breast cancer in July 2019.

### 2.3. Second Case

Patient No. 2, a 36-year-old woman, developed triple-negative right breast cancer (invasive ductal carcinoma, stage T2N0M0) in October 2015. Serum CA15-3 was 21 IU/mL (normal <28 IU/mL). She underwent breast-conserving surgery followed by adjuvant chemotherapy (TAC: docetaxel 75 mg/m^2^, cyclophosphamide 500 mg/m^2^, and doxorubicin 50 mg/m^2^). Because her grandmother had a history of breast cancer, she underwent BRCA1 and BRCA2 testing. Sequencing analysis showed a deletion at position 922–924 of BRCA1 that generated a stop codon (p.Lys307_Ser308insTer). A mutation was found in BRCA2 at the amino acid position 1200 (p.Asp1199_Cys1200insTer) (Table 2). Four years of routine follow-up revealed no evidence of disease. She did not receive any additional preventive mastectomies or undergo ovarian resection. 

### 2.4. Third Case

Patient No. 3, a 31-year-old woman, was diagnosed with bilateral breast cancer (right invasive ductal carcinoma and left ductal carcinoma in situ) in October 2011. Serum CA15-3 was 23 IU/mL (normal <28 IU/mL). After a bilateral mastectomy, pathologic staging revealed T2N0M0 for the right breast cancer and pathologic stage TisN0 for the left breast cancer. Both tumors were estrogen receptor (ER)-positive, progesterone receptor (PR)-positive, and HER2 negative. This patient was treated with adjuvant chemotherapy (FAC: 5-fluorouracil 500 mg/m^2^, doxorubicin 50 mg/m^2^, cyclophosphamide 500 mg/m^2^). Because of bilateral breast cancer, she underwent BRCA1 and BRCA2 testing. A frame shift mutation was found in BRCA1 at the amino acid position 1833 (p.Val1833fs). Sequence analysis showed a base substitution at position 7480 of BRCA2 that generated a stop codon (p.Arg2494Ter) (Table 2). This patient underwent preventive bilateral salpingo-oophorectomy and has been taking tamoxifen as adjuvant hormone therapy for 8 years. There have been no recurrences of breast cancer over 96 months of follow-up.

### 2.5. Fourth Case

Patient No. 4, a 35-year-old woman, developed left breast cancer (triple-negative) in October 2003. Serum CA15-3 was 17 IU/mL (normal <28 IU/mL). She underwent left breast-conserving surgery followed by radiotherapy and adjuvant chemotherapy for pT1N0M0 (FAC: 5-fluorouracil 500 mg/m^2^, doxorubicin 50 mg/m^2^, cyclophosphamide 500 mg/m^2^). In September 2009, she developed triple-negative right breast cancer. After partial mastectomy of the right breast cancer, the pathologic stage of the breast cancer was found to be stage I (ypT1N0M0). A frame shift mutation was found in BRCA1 at the amino acid position 1677 (p.Thr1677fs). Sequence analysis showed a base substitution at position 1399 of BRCA2 that generated a stop codon (p.Lys467Ter) (Table 2). She underwent preventive salpingo-oophorectomy in 2017. There was no recurrence of breast cancer over a follow-up period of 126 months (Table 3).

### 2.6. Clinical Characteristics of Patients with a Pathogenic gBRCA1 Mutation and gBRCA2 Variants of Uncertain Significance 

There were seven patients (7/2720; 0.25%) who had a pathogenic gBRCA1 mutation and gBRCA2 variants of uncertain significance (VUS), but no patients who had a pathogenic gBRCA2 mutation and gBRCA1 VUS. All patients with pathogenic gBRCA1 and gBRCA2 VUS were female and had a mean age at diagnosis for breast cancer of 32 years (range, 28–36 years). All seven tumors were infiltrating ductal carcinomas. Six patients received breast-conserving surgery, while the seventh patient received total mastectomy. Pathologic stage was II in four tumors, I in two tumors, and III in one tumor. Most of the patients (*n* = 5) underwent BRCA mutation testing because of their young age (age <40). Immunohistochemistry (IHC) revealed that five of the patients had triple-negative breast cancer (*n* = 5, 71.4%). One patient had an ER-negative, PR-weak positive, and HER2-negative tumor, while the other patient had an ER-positive, PR-positive, and HER2-positive tumor. Clinical characteristics of these patients are described in Table 1.

**Figure 5:**
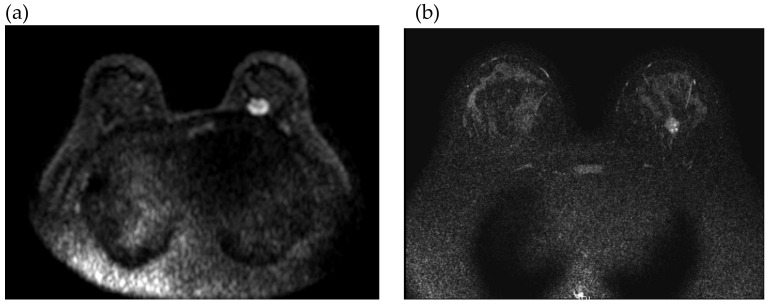
Imaging of the breasts on magnetic resonance imaging (MRI). (**a**) Breast MRI of patient No. 6 at the first diagnosis of breast cancer in October 2013. (**b**) Breast MRI of patient No. 6’s sister at the first diagnosis of breast cancer in January 2016.

One of these seven patients (No. 6) had a family history of breast cancer (Figure 5). This patient, who had left breast cancer (ER-negative, PR-positive, and HER2-negative invasive ductal carcinoma) was diagnosed at age 28 in October 2013. She underwent left breast-conserving surgery (pT2N1M0) followed by radiotherapy and adjuvant chemotherapy. She was subsequently treated at another hospital, and lost to follow-up. A frame shift mutation was found in BRCA1 at the amino acid position 3627 (p.Glu1210Argfs). Sequence analysis showed a base substitution at position 5969 of BRCA2 that generated mutation at the amino acid position 1990 (p.Asp1990Ala) (Table 2). Her sister, who was diagnosed with left breast cancer (TNBC, invasive ductal carcinoma) at age 35 in 2016, underwent left breast-conserving surgery (pT1N0M0) followed by radiotherapy and adjuvant chemotherapy (TC). Interestingly, the sister had the same BRCA1 mutation as patient No. 6, c.3627dupA (p.Glu1210Argfs), but no BRCA2 mutation (Table 4). 

One (No. 7) patient had a BRCA1 mutation and three BRCA2 VUS. A frame shift mutation was found in BRCA1 at the amino acid position 3627 (p.Glu1210Argfs). Sequence analysis showed the base substitutions at position 1362 (P.Lys454=), 2127 (p.Leu709=), and 5785 (p.Ile1929Val) of BRCA2 (Table 2). Her BRCA2 VUSs were later determined to be benign according to review by an expert panel. She had right breast cancer (triple-negative invasive ductal carcinoma, stage T1N0M0) that was diagnosed at age 28 in November 2014. She underwent breast-conserving surgery followed by adjuvant chemotherapy (FAC: 5-fluorouracil 500 mg/m^2^, doxorubicin 50 mg/m^2^, cyclophosphamide 500 mg/m^2^), and radiotherapy. Her grandmother had ovarian cancer. During 5 years of follow-up, no recurrence of breast cancer was observed. 

One (No. 9) out of these seven patients had cancer recurrence. This patient had right breast cancer (triple-negative invasive ductal carcinoma, stage T1N1M0) and was diagnosed at age 35 in April 2016. Sequence analysis showed a base substitution of BRCA1 (c.302-2A>C). Sequence analysis showed the base substitutions at position 1568 of BRCA2 (p.His523Arg) (Table 2). She underwent neoadjuvant chemotherapy (doxorubicin and cyclophosphamide followed by docetaxel) followed by breast-conserving surgery and adjuvant radiotherapy. Three years after surgery, the disease recurred in her left breast. In September 2019, total mastectomy of the left breast and prophylactic right mastectomy were performed. The pathologic diagnosis of the left breast cancer was a triple-negative invasive ductal carcinoma (pT1N0M0). She underwent palliative chemotherapy (paclitaxel and carboplatin) for four cycles. The patient rejected chemotherapy and is under observation; her progression-free survival (PFS) is 5 months over a 41 month follow-up period. 

## 3. Discussion

All four patients with both gBRCA1 and gBRCA2 mutations had mutations that have been described previously; However, double BRCA1 and BRCA2 mutations are very rare [7,8]. We found that patients who carry gBRCA1 and gBRCA2 mutations are very rare and are diagnosed with breast cancer at a young age. 

In both gBRCA1 and gBRCA2, mutations in coding areas were distributed evenly along the length of each gene in approximate proportion to the relative size of the exons [9]. TNBCs are enriched for HRR gene defects, and there is evidence that somatic alterations in TNBCs share molecular features of ovarian cancer, including an elevated level of genomic instability and specific chromosomal gains on 1q, 3q, 8q, and 12p and losses on 4q, 5q, and 8p [10]. BRCA1 and BRCA2 mutated breast cancers have been reported to have different molecular characteristics from each other, for example, a correlation between TNBC and BRCA1 but not BRCA2 [11]. Breast cancer cells with deleterious mutations in BRCA1/2 are deficient in the repair mechanism for DNA double-strand breaks, leaving these tumors highly dependent on the repair pathway for single-strand breaks [12]. In Asian countries, including Korea, the most common BRCA1 mutation is 185delAG (c.68_69delAG), and the most common BRCA2 mutation is BRCA2 c.7480C > T [13].

BRCA1 plays a major role in DNA repair through homologous recombination (HR). While BRCA2 is directly involved in RAD51 protein-mediated repair, BRCA1 appears to act via a more complex mechanism through interaction with other proteins [14]. The question then becomes how BRCA1 and BRCA2 mutations lead to genetic instability? BRCA1 mutation carriers develop breast and ovarian cancer at a younger age than BRCA2 mutation carriers, and BRCA2 is associated with fewer cases of breast cancer than BRCA1 [15]. Three of the four patients who had germline mutations in both BRCA1 and BRCA2 were TNBC patients. Most patients with pathogenic gBRCA1 mutation and gBRCA2 VUS also had TNBC. Given that a large proportion of BRCA1-mutated tumors are TNBC, we hypothesize that BRCA1 plays a leading role in the clinical presentation of patients who carry germline mutations in both BRCA1 and BRCA2 [16]. In the previous study, more TNBC cases were found to be associated with BRCA1 mutation than BRCA2 mutation [13]. It is likely that mutations in both gBRCA1 and gBRCA2 genes increase the risk for cancer development, possibly at a younger age [17].

There were no patients with gBRCA2 mutation and gBRCA1 VUS. Given this, we hypothesize that gBRCA2 mutations have different functional consequences than gBRCA1 mutations. gBRCA2 mutations increase the lifetime risk of breast cancer development in women by 50–60%, whereas the lifetime risk of breast cancer in women with gBRCA1 mutations is 70–80% and the lifetime risk of ovarian cancer is 50% [18]. Further research is needed to determine the roles of gBRCA1 and gBRCA1 and their interaction.

Hereditary Breast and Ovarian Cancer Syndrome (HBOC) is considered when there are multiple cases of breast cancer and/or ovarian cancer on the same side of the family. Patient No. 6 had breast cancer with gBRCA1 mutation (c.3627dupA), while her sister had breast cancer with the same gBRCA1 mutation (c.3627dupA). Both sisters had breast cancer on the left side, but one cancer was TNBC while the other was hormone receptor-positive breast cancer. This indicates that not only genetic factors but also other factors determine breast cancer phenotype.

This study had several limitations. First, BRCA mutation testing was performed by direct sequencing of all exons and flanking intronic sequences. The sensitivity of this test is known to be between 60% and 70% and cannot identify large genomic rearrangements. Additional tests to verify the deletion/duplication of BRCA genes require multiplex ligation-dependent probe amplification (MLPA). MLPA test for BRCA mutation is recommended for breast cancer patients with a familial history of breast and/or ovarian cancer, young breast cancer patients, and patients who test negative for BRCA1/2 small mutations in initial testing [19]. There were no patients who had MLPA testing performed in this study, and thus we may have missed some patients with BRCA mutations. This could have resulted in the underestimation of the prevalence of BRCA mutation. Additionally, no patients were treated with poly-adenosine diphosphate-ribose polymerase (PARP) inhibitors. In a previous study, BRCA carriers who had ovarian, breast, or prostate cancers benefitted clinically from treatment with Olaparib [20]. Lastly, the mechanism linking double gBRCA1/2 mutation with the phenotype of TNBC is unclear.

## 4. Materials and Methods

A retrospective review of medical records was done to identify breast cancer patients with gBRCA mutation who underwent surgery at Samsung Medical Center (SMC) between January 2007 to October 2018. This research was approved by the institutional review board (IRB) of SMC (SMC 2019-03-067). Informed consent for genomic analysis was obtained from all patients. All samples were obtained with the approval of the IRB (SMC 2019-03-067). For gBRCA testing, genomic DNA was extracted from peripheral blood leukocytes and sequenced by direct Sanger sequencing (total of 22 exons). Direct Sanger sequencing annotation was performed based on a pre-defined internal calling algorithm. Pathogenic mutations in gBRCA1 and gBRCA2 genes were investigated in this study in addition to other genetic VUS.

BRCA1 and BRCA2 are on chromosomes 17q21.31 and 13q12.3, respectively. The results of BRCA testing were reported accordingly to the American College of Medical Genetics (ACMG) standards and guidelines [21], Korea ONCOgene Research and Diagnosis (KONCORD) [22], and multifactorial probability-based model of IARC (International Agency for Research on Cancer) [23]. Mutations were classified based on the ClinVar database maintained by the National Center for Biotechnology Information (NCBI) [24]. The mutations or VUS are reported according to the HGVS (Human Genome Variation Society) [25].

The following variables were extracted from clinical records: age at diagnosis, sex, reason for BRCA testing, marriage, parous status, family history of breast cancer or ovarian cancer, menopause status, surgical record, histopathology, and pathologic stage. Date of last follow-up or death, survival status, cause of death (if applicable), and relapse status were collected. Progression-free survival (PFS) was defined as the time from the date of first-line palliative chemotherapy to the progression of cancer or death from any cause. Overall survival (OS) was defined as the time from the date of surgery to the date of the last follow-up or of death from any cause. ER and PR expression were measured by IHC. TNBC was defined as both ER and PR negative and lacking overexpression of HER2. 

## 5. Conclusions

To the best of our knowledge, this is the first case series of Korean breast cancer patients with both gBRCA1 and gBRCA2 mutations based on a single-center experience. Given the difference in the prevalence of gBRCA1/2 according to the ethnic group, further multicenter research is needed to identify the characteristics of breast cancer patients who carry pathogenic mutations in both gBRCA1 and gBRCA2.

## Figures and Tables

**Table 1:** Clinical characteristics of patients with mutations in both gBRCA1 and gBRCA2 and patients with gBRCA1 mutation and gBRCA2 VUS.

Characteristic		BRCA1 and BRCA2 (N = 4)	BRCA1 and BRCA2 VUS (N = 7)
Age at diagnosis (year)	Mean (range)	36 (31–43)	32 (28–36)
Sex	Female Male	4 0	7 0
Reason for BRCA test	Family history of breast cancer Family history of ovarian cancer Bilateral breast cancer Age < 40 years	4 1 2 3	1 2 0 5
Married	Yes No	3 1	3 4
Menopausal status	Premenopausal	4	7
Family history of cancer	Breast Ovary Other	3 1 0	2 2 4
Prophylactic mastectomy	Yes No	3 1	3 4
Prophylactic oophorectomy	Yes No	3 1	0 7
Surgical record	TM BCS	2 2	1 6
TNM stage	I II III	1 3 0	2 4 1
Pathology	Invasive ductal carcinoma	4	7
IHC subtype	ER (−)/PR (+)/HER2 (−) ER (+)/PR (+)/HER2 (−) ER (+)/PR (+)/HER2 (+) ER (−)/PR (−)/HER2 (−)	0 1 0 3	1 0 1 5

TM, total mastectomy; BCS, breast-conserving surgery; IHC, immunohistochemistry; ER, estrogen receptor; PR, progesterone receptor; HER2, human epidermal growth factor receptor 2; VUS, variant of uncertain significance.

**Table 2:** Genetic mutations in patients with pathogenic mutations in both gBRCA1 and gBRCA2 and patients with gBRCA1 mutation and gBRCA2 VUS.

No.	Age at Diagnosis	Gene	Mutation	Pathogenic
1	43	BRCA1 BRCA2	c.390C > A (p.Tyr130Ter) c.5576_5579del (p.Ile1859fs)	P P
2	36	BRCA1 BRCA2	c.922_924delinsT (p.Lys307_Ser308insTer) c.3599_3600del (p.Asp1199_Cys1200insTer)	P P
3	31	BRCA1 BRCA2	c.5496_5506delinsA (p.Val1833fs) c.7480C > T (p.Arg2494Ter)	P p
4	35	BRCA1 BRCA2	c.5030_5033del (p.Thr1677fs) c.1399A > T (p.Lys467Ter)	P P
5	34	BRCA1 BRCA2	c.5445G > A (p.Trp1815Ter) c.10150C > T (p.Arg3384Ter)	P VUS
6	28	BRCA1 BRCA2	c.3627dupA (p.Glu1210Argfs) c.5969A > C (p.Asp1990Ala)	P VUS
7	28	BRCA1 BRCA2 BRCA2 BRCA2	c.3627dupA (p.Glu1210Argfs) c.1362A > G (P.Lys454=) c.2127G > C (p.Leu709=) c.5785A > G (p.Ile1929Val)	P VUS → B VUS → B VUS → B
8	36	BRCA1 BRCA2	c.4933delA (p.Arg1645Glyfs) c.8187G > T (p.Lys2729Asn)	P VUS → B
9	35	BRCA1 BRCA2	c.302-2A > C c.1568A > G (p.His523Arg)	P VUS
10	30	BRCA1 BRCA2	c.2405_2406delTG (p.Val802Glufs) c.8187G > T (p.Lys2729Asn)	P VUS → B
11	35	BRCA1 BRCA2	c.5080G > T (p.Glu1694Ter) c.5969A > C (p.Asp1990Ala)	P VUS

P, pathogenic; VUS, variant of uncertain significance; B, benign. The mutations or VUS are reported according to the HGVS (Human Genome Variation Society).

**Table 3:** Treatment outcomes of patients with pathogenic mutations in both gBRCA1 and gBRCA2 and patients who had a gBRCA1 mutation and gBRCA2 VUS.

No.	NeoadjuvantCTx.	Adjuvant Therapy	Recurrence	Palliative CTx.	OS (Months)
1	AC + D	RTx.	Yes	Yes (TC)	42
2	-	CTx. (TAC) + RTx.	No	No	48
3	-	CTx. (FAC) + Tamoxifen	No	No	96
4	-	-	No	No	126
5	AC + D	-	No	No	42
6	-	-	F/U loss	-	3
7	-	CTx. (FAC) + RTx.	No	-	62
8	AC	CTx. (docetaxel and Herceptin) + Tamoxifen + RTx.	No	-	48
9	AC + D	RTx.	Yes	Yes (TC)	39
10	AC + D	RTx	No	-	40
11	AC + D	RTx.	No	-	40

CTx.: chemotherapy; RTx.: radiotherapy; VUS, variant of uncertain significance; AC + D: doxorubicin and cyclophosphamide followed by docetaxel; TAC: docetaxel, cyclophosphamide, and doxorubicin; FAC: 5-fluorouracil, doxorubicin, and cyclophosphamide; TC: paclitaxel, carboplatin; OS: overall survival.

**Table 4:** Family history of patients with pathogenic mutations in both gBRCA1 and gBRCA2 and patients who had a gBRCA1 mutation and gBRCA2 VUS.

No.	Parous Status	Family History	No. of Relatives with Cancer	Type of Relative with Breast/Ovarian Cancer	Remarks
1	P	Breast cancer	1	Mother only	-
2	P	Breast cancer	1	Grandmother only	-
3	P	Breast cancer	1	Sister only	-
4	N	Ovarian cancer	1	Grandmother only	-
5	N	-	-	-	-
6	N	Breast cancer Ovarian cancer	1 1	Sister only Aunt only	gBRCA1m -
7	N	Ovarian cancer Cervical cancer Colon cancer	111	Grandmother only - -	-
8	P	Hepatocellular carcinoma	1	-	-
9	P	Gastric cancer	1	-	-
10	N	Colon cancer	1	-	-
11	P	Breast cancer	1	Mother only	-

gBRCA1m, germline breast cancer susceptibility gene 1 mutation; P, parous; N, nulliparous; VUS, variant of uncertain significance.
